# Validation of the 8th Edition American Joint Commission on Cancer (AJCC) Gallbladder Cancer Staging System: Prognostic Discrimination and Identification of Key Predictive Factors

**DOI:** 10.3390/cancers13030547

**Published:** 2021-02-01

**Authors:** Dimitrios Giannis, Marcelo Cerullo, Dimitrios Moris, Kevin N. Shah, Garth Herbert, Sabino Zani, Dan G. Blazer, Peter J. Allen, Michael E. Lidsky

**Affiliations:** 1 Institute of Health Innovations and Outcomes Research, The Feinstein Institutes for Medical Research, Northwell Health, Manhasset, NY 11030, USA; dimitrisgiannhs@gmail.com; 2 Department of Surgery, Duke University Medical Center, Durham, NC 27710, USA; marcelo.cerullo@duke.edu (M.C.); kevin.n.shah@duke.edu (K.N.S.); garth.herbert@duke.edu (G.H.); sabino.zani@duke.edu (S.Z.); trey.blazer@duke.edu (D.G.B.3rd); peter.allen@duke.edu (P.J.A.); michael.lidsky@duke.edu (M.E.L.)

**Keywords:** gallbladder cancer, AJCC, National Cancer Database, staging

## Abstract

**Simple Summary:**

Despite implementing numerous changes in the American Joint Committee on Cancer (AJCC) staging system for gallbladder cancer (GBC), the ability to accurately prognosticate survival in these patients has not been vigorously evaluated. The purpose of our study was to compare the prognostic ability of AJCC 7th and 8th edition, investigate the effect of AJCC 8th edition nodal status on the survival, and identify risk factors associated with the survival after N reclassification in GBC patients. We used the largest cancer database in the United States and determined that the updated AJCC 8th edition GBC staging system was comparable to the 7th edition, with no major improvements in survival discrimination. The recently implemented changes in N classification do not appear to improve the prognostic performance of the AJCC cancer staging system with regard to survival in GBC patients.

**Abstract:**

The scope of our study was to compare the predictive ability of American Joint Committee on Cancer (AJCC) 7th and 8th edition in gallbladder carcinoma (GBC) patients, investigate the effect of AJCC 8th nodal status on the survival, and identify risk factors associated with the survival after N reclassification using the National Cancer Database (NCDB) in the period 2005–2015. The cohort consisted of 7743 patients diagnosed with GBC; 202 patients met the criteria for reclassification and were denoted as stage ≥III by AJCC 7th and 8th edition criteria. Overall survival concordance indices were similar for patients when classified by AJCC 8th (OS c-index: 0.665) versus AJCC 7th edition (OS c-index: 0.663). Relative mortality was higher within strata of T1, T2, and T3 patients with N2 compared with N1 stage (T1 HR: 2.258, *p* < 0.001; T2 HR: 1.607, *p* < 0.001; Τ3 HR: 1.306, *p* < 0.001). The risk of death was higher in T1–T3 patients with Nx compared with N1 stage (T1 HR: 1.281, *p* = 0.043, T2 HR: 2.221, *p* < 0.001, T3 HR: 2.194, *p* < 0.001). In patients with AJCC 8th edition stage ≥IIIB GBC and an available grade, univariate analysis showed that higher stage, Charlson–Deyo score ≥ 2, higher tumor grade, and unknown nodal status were associated with an increased risk of death, while year of diagnosis after 2013, academic center, chemotherapy. and radiation therapy were associated with decreased risk of death. Chemotherapy and radiation therapy were associated with decreased risk of death in patients with T3–T4 and T2–T4 GBC, respectively. In conclusion, the updated AJCC 8th GBC staging system was comparable to the 7th edition, with the recently implemented changes in N classification assessment failing to improve the prognostic performance of the staging system. Further prospective studies are needed to validate the T2 stage subclassification as well as to clarify the association, if any is actually present, between advanced N staging and increased risk of death in patients of the same T stage.

## 1. Introduction

Primary gallbladder carcinoma (GBC) is a rare, yet often lethal biliary tract malignancy with an incidence of up to 12,190 new cases annually and 1–2.5 cases per 100,000 population at risk in the United States [1,2,3]. The incidence of GBC is higher in females; white population; and in some geographical areas, such as South America, India, and Japan [4,5,6,7]. GBC incidence increases with advanced age, with a median age at diagnosis of 69 years [7]. Additional risk factors commonly implicated in the pathogenesis of GBC include cholelithiasis, chronic biliary tract infection (*Salmonella typhi*, *Helicobacter species*), tobacco use, prolonged fertility, obesity, primary sclerosing cholangitis, and exposure to metals and metalloids including arsenic [8,9,10,11,12,13,14]. 

GBC cancer is diagnosed incidentally in approximately 50% of the cases, and 3 out of 4 patients undergoing re-exploration have residual disease [15]. For the remainder of patients, diagnosis of GBC is usually made at advanced stages owing to obscure clinical manifestations and the absence of effective screening modalities. Furthermore, malignant cells often invade the liver early in the disease process because of the absence of serosa [2,16,17], which generally portends a worse prognosis. Owing to aggressive biology and often late presentation, prognosis is poor, with a median survival of 6 months and a 5-year overall survival (OS) rate less than 10% for all patients [1,16,18,19,20].

Effective treatment hinges on complete resection of the tumor, ranging from simple cholecystectomy to major hepatectomy and portal lymphadenectomy [21], combined with effective cytotoxic therapy [22,23]. Nevertheless, relatively few patients are eligible for surgical resection. Hawkins et al. reported that 34% of GBC cases present with jaundice, which is an ominous sign of advanced nonresectable disease [24], in the context of 59% already presenting with advanced disease. Patients with locally advanced or metastatic GBC are treated with gemcitabine plus cisplatin based on the survival benefit demonstrated in the ABC-02 trial, but prognosis remains exceedingly poor, with a median survival less than one year [25]. For patients eligible for curative intent resection, adjuvant chemotherapy was recently demonstrated to improve survival. The BILCAP trial showed that adjuvant capecitabine improves overall survival by 17 months in surgically resected biliary tract cancer patients, including GBC (17.7% of the trial population vs. 82.3% with cholangiocarcinoma) [22]. 

Appropriate counseling of GBC patients regarding prognosis and available treatment options requires accurate staging criteria. The Union for International Cancer Control (UICC) and American Joint Committee on Cancer (AJCC) developed the TNM staging system in order to evaluate disease progression, predict survival, and facilitate appropriate treatment planning. Since its first published edition in 1977, this tumor classification system has been modified and updated regularly through the supplementation of new data [26]. The most recent 8th edition of the TNM classification was published in 2016, in which the T and N staging was re-classified from the 7th edition published in 2010 [27] (Table 1). Based on the findings of the study by Shindoh et al., T2 stage is now subdivided as T2a (peritoneal side tumor location) or T2b (hepatic side tumor location), considering that hepatic side localization has been reported to portend a worse prognosis [28,29]. This is of clinical importance as an international multicenter study showed that the T2 tumor location (peritoneal vs hepatic) in patients treated with radical resection effectively predicts survival, revealing a higher 5-year OS in T2a compared with T2b patients (75.5% vs. 48.2%, respectively) [29]. Furthermore, N classification has been modified to assess the number of lymph nodes involved, instead of the previously used location-based evaluation. At least six nodes should be evaluated in order to adequately classify patients as N0 (no regional nodes), N1 (1–3 metastatic regional nodes), or N2 (≥4 metastatic regional nodes) [28].

Despite implementing numerous changes in the AJCC 8th edition staging system, their performance on correctly classifying patients according to their survival has not been vigorously evaluated. The objective of our study was to assess the performance of AJCC 8th versus 7th edition, investigate the effect of AJCC 8th edition nodal status classification on the survival of patients with different T stage, and identify factors associated with the survival of GBC patients after N reclassification using GBC patient data derived from the National Cancer Database (NCDB).

## 2. Results

### 2.1. NCDB Cohort AJCC 8th and 7th Classification and Comparison (Overall Survival c-Index)

**Figure 1:**
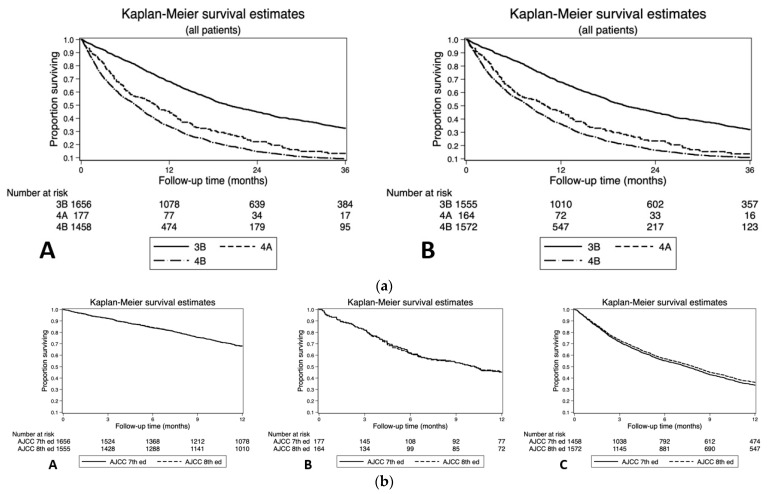
(**a**) Overall survival (OS) of reclassified patients according to the (A) American Joint Committee on Cancer (AJCC) 7th and (B) 8th editions TNM classification. (**b**) Overall survival (OS) pairwise comparison of (A) stage 3B, (B) stage 4A, and (C) stage 4B reclassified patients according to the AJCC 7th and 8th editions TNM classification.

The NCDB cohort consisted of 7743 patients diagnosed with GBC at a mean age of 69.7 years (SD: 12.2). In total, 7541 patients had no change in their staging between the AJCC 8th and 7th editions (Table 2a,b), while 202 patients (2.61%) met the criteria for reclassification and were denoted as stage III or above by AJCC 7th edition criteria (Figure 1a). Pairwise comparison of one-year overall survival (OS) between the AJCC 7th and AJCC 8th editions revealed similar results for stage IIIB (68.1%, [95% confidence interval (CI): 65.8–70.3%] versus 67.8% [95% CI: 65.4–70.1%]), stage IVA (45.2%, [95% CI: 37.7–52.3%] versus 45.8% [95% CI: 38.0–53.3%]), and stage IVB patients (33.8% [95% CI: 31.3–36.2%] versus 36.2% [95% CI: 33.8–38.6%], Figure 1b). Overall survival concordance indices were similar for patients when classified by AJCC 8th (OS c-index: 0.665) versus AJCC 7th edition (OS c-index: 0.663).

### 2.2. Impact of Nodal Disease on Survival of GBC Patients after AJCC 8th T-Stage Reclassification

**Figure 2:**
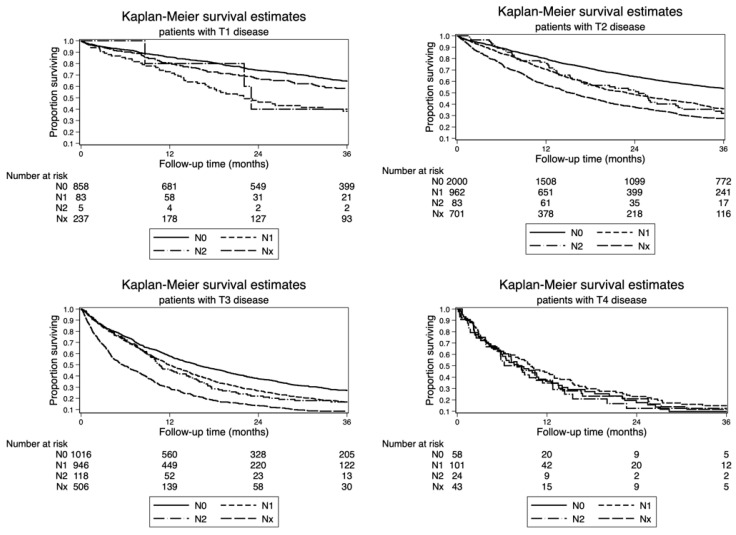
Impact of nodal disease on overall survival (OS) stratified by T-stage (Kaplan–Meier method) according to the AJCC 8th edition TNM classification.

The association between nodal status classification and AJCC 8th edition T stage is depicted in Figure 2. Relative mortality was higher within strata of T1, T2, and T3 patients with N2 compared with N1 stage (T1 HR: 2.258, *p* < 0.001; T2 HR: 1.607, *p* < 0.001; Τ3 HR: 1.306, *p* < 0.001; Table 3). N2 nodal disease was not identified as a significant factor of increased risk of death in T4 patients. The risk of death was higher in T1–T3 patients with Nx compared with N1 stage (T1 HR: 1.281, *p* = 0.043, T2 HR: 2.221, *p* < 0.001, T3 HR: 2.194, *p* < 0.001).

### 2.3. Factors Associated with Risk of Death in GBC Patients Stratified by AJCC 8th T-Stage Classification

Regression analysis yielded associations with GBC-specific mortality, stratified by T stage using the AJCC 8th edition classification (Table 4).

#### 2.3.1. T1 Stage

The following factors were associated with increased risk of death: age ≥ 70 years, Charlson–Deyo score ≥ 2, higher tumor grade, and N1 stage. Female sex and year of diagnosis after 2010 were associated with decreased risk of death.

#### 2.3.2. T2 Stage

The following factors were associated with increased risk of death: age ≥ 70 years, black race, higher Charlson–Deyo score, non-private insurance, higher tumor grade, and higher N stage. Race other than black or white, academic center, higher center volume, radiation therapy, and year of diagnosis after 2010 were associated with decreased risk of death.

#### 2.3.3. T3 Stage

The following factors were associated with increased risk of death: Charlson–Deyo score = 1, non-private insurance, higher tumor grade, and higher N stage. Race other than black or white, year of diagnosis after 2010, academic center, chemotherapy, and radiation therapy were associated with decreased risk of death.

#### 2.3.4. T4 Stage

Chemotherapy and radiation therapy were associated with decreased risk of death.

### 2.4. Factors Associated with Risk of Death in GBC Patients with Stage ≥ IIIB Classified by AJCC 8th Edition

The following factors were associated with increased risk of death: higher stage, Charlson–Deyo score ≥2, higher tumor grade, and unknown nodal status (Table 5). Year of diagnosis after 2013, academic center, chemotherapy, and radiation therapy were associated with decreased risk of death.

## 3. Discussion

Our analysis showed that the OS concordance indices were similar for patients when classified by AJCC 8th versus AJCC 7th edition. Furthermore, comparative analysis between the AJCC 8th to 7th editions in patients with ≥IIIB stage GBC revealed a similar 1-year OS in stage IIIB, stage IVA, and stage IVB patients. While we were not able to evaluate changes to the T2 stage subclassification in the AJCC 8th edition owing to limitations of the dataset used, these data do demonstrate that the updated N stage criteria does not improve prognostic discrimination in GBC. These findings are concordant with the NCDB analysis by Oweira et al. [30] and SEER analysis by Jiang et al. [31], but were different from the OS c-index reported by Lee et al. (AJCC 8th OS c-index = 0.832, AJCC 7th OS c-index = 0.845) [32]. These differences could be attributed to the different methodologic approach followed by Lee et al. and their smaller sample size of 2800 patients in their AJCC 8th survival analysis [32].

Analysis of N status stratified by T stage in the AJCC 8th edition showed that relative mortality was higher in patients with T1, T2, and T3 disease with N2 compared with N1 disease, whereas N2 nodal disease was not identified as a significant factor of increased risk of death in T4 patients. In addition, the risk of death was higher in T1–T3 patients with Nx compared with N1 stage, suggesting that inadequately staged patients, owing to a lack of nodal analysis, may actually have had more advanced disease. Despite this finding, the N classification appears to be of less relevance in patients with T4 stage. Of interest, diagnosis after 2010 was associated with decreased risk of death in patients with T1–T3 disease, while academic center facility type and race other than white or black were associated with decreased risk of death in T2–T3 patients. The management of GBC at an academic center has been previously associated with a higher median OS compared with management at a community center (21.0 vs. 17.7 months, respectively, *p* = 0.002) [33], and our data further support this finding. Multidisciplinary approaches to patient care, adherence to guidelines, earlier adoption of adjuvant chemotherapy, and disease-specific subspecialist expertise may potentially explain the observed differences, but further research is warranted. Regarding race, previous studies have shown that black patients are at increased risk of death compared with white patients [34], and black and Hispanic patients are less likely to receive curative intent surgery with adequate lymph node dissection compared with white patients [35]. In our study, black patients with T2 stage disease were at increased risk of death, and these findings should be further investigated to elucidate any association or confounding factors related to treatment disparities between races.

Chemotherapy and radiation were associated with decreased risk of death in patients with T3–T4 and T2–T4 disease, respectively. The recently published BILCAP trial, of which 17.7% of the total trial population had GBC, demonstrated that adjuvant chemotherapy improves survival in surgically resected patients by 17 months [22]. The beneficial effect of radiation on the survival of GBC patients seen in our data is supported by some single institution studies [36,37,38,39,40], while previous SEER studies have reported conflicting results [34,41]. This is of clinical relevance and warrants further investigation regarding the role of radiation therapy in the management of GBC.

In the United States (US), approximately 12,190 new cases of GBC are diagnosed and 3790 patients die annually [3]. Complete tumor resection is considered to be the only potentially curative modality of treatment in patients presenting with resectable tumors [42,43]. The role of radiation and chemotherapy is still under investigation, with recent data suggesting a possible association with improvement in survival [44,45], including the data presented herein. Accurate staging is of extreme importance, not only for selecting appropriate treatment and follow-up plans, but also to predict survival. The American Joint Committee on Cancer (AJCC) remains the most commonly utilized cancer-stratification system, which is periodically reviewed and updated upon current data availability. AJCC TNM staging is expected to have moderate discriminatory potential as it is based on a small number of variables in order to remain simple and comprehensible [46]. The 8th edition of the AJCC Staging System was released in 2016, and it was recommended as a replacement for the previous version. AJCC 8th gallbladder cancer staging includes changes in T (T2 stage subdivision now includes T2a/peritoneal and T2b/hepatic side tumor location) and N (evaluation of metastatic lymph node number instead of location). The newly integrated parameters of T and N staging were based on data reported from multiple centers worldwide [27,28,30]. Only a few studies with large databases have validated the AJCC 8th TNM classification for GBC [30,32]. Largely, the overall performances of the AJCC 8th and AJCC 7th systems were comparable when applied to patients in the NCDB, suggesting that recent N staging implementations did not have a great impact in the overall discrimination of GBC patients. This is highlighted by the limited prognostic utility of AJCC 8th due to the inaccurate stratification of N2 disease and the aberrant survival reversal of IIIA and IIIB patients. Consistent with this finding, Wang et al. also reported that stage IIIA patients, surprisingly, had poorer survival than stage IIIB patients [47]. Naturally, patients with T3N0M0 disease are always expected to have a more favorable survival than those with T3N1M0. However, studies using SEER and NCDB data have observed that patients with T3N0M0 or T3N1M0 disease had similar survival, which was poorer than that of patients with T1–2N1M0 disease.

As this paradox was also noted in the AJCC 7th edition, it might imply that the changes in N classification alone are not the main factor of these outcomes. The lymph node status is undoubtedly a significant prognostic factor in patients with GBC. However, based on the comparable classification accuracy between the AJCC 8th (number-based) and 7th (location-based) classification systems, it seems that the optimal method of lymph node stratification has yet to be determined. Population-based studies provided the information that supported the implementation of a lymph node number-based approach with or without the evaluation of the lymph node ratio [30,48,49,50]. This is of paramount clinical importance, especially for patients with pT1bN0 and pT2N0 GBC, as it may impact treatment recommendations [51]. Of interest, a recent study comparing data from SEER and China showed that, despite the fact that all N2 diseases are grouped into stage IVB in the AJCC 8th edition, some patients with N2 disease could undergo R0 resection that involves regional lymph nodes routinely removed during radical GBC resection, and had longer survival than patients with M1 disease. This might imply that selected patients with advanced GBC can benefit from surgical resection.

Current data regarding positive lymph node number (PLNN) or ratio (PLNR) utility on the estimation of GBC patients’ survival vary. Liu et al. have identified total lymph nodes (TLNs) as well as a positive disease lymph node ratio (PLNR) as strong and independent predictors of disease-specific survival in GBC patients undergoing curative intent surgery [49]. Interestingly, Negi et al. showed that PLNR is a significant prognostic factor of disease-free survival, whereas location of positive lymph nodes was not associated with prognosis [48]. Nevertheless, Shirai et al. in their retrospective study reported that PLNN is a better predictor of GBC prognosis compared with location or PLNR [52]. This NCDB analysis demonstrated an expected finding of increased risk of death in T1N2, T2N2, and T3N2 compared with T1N1, T2N1, and T3N1 respectively.

The NCDB has been previously analyzed to investigate cancer risk factors, treatment, and survival in the U.S. population. The NCDB remains a valuable resource for evaluating patient-related and hospital-related factors that may impact patient care and oncologic outcomes, particularly in patients with rare malignancies such as GBC. The NCDB is a unique clinical database in that it collates both demographic and oncology-specific information, the extent of surgery, and margin positivity. While analysis of GBC patients in the NCDB provided us with increased study power considering the large sample size of patients, there are several limitations that affect the validity of our findings. Our analysis is retrospective and the quality of the data is affected by the variables provided by the NCDB. The AJCC 8th edition further classifies patients with T2 tumors into T2a and T2b stage. Unfortunately, the NCDB does not provide adequate granularity regarding the new T2 subclassification at this time, and we were there thus not able to corroborate or refute previous findings. In addition, the NCDB does not discriminate between one-stage and two-stage resections, which may impair data reporting accuracy, including the rate of lymphadenectomies in patients with incidental GBC. Lower numbers of resected lymph nodes or variability between pathologic reports may have affected the number of positive lymph nodes reported in NCDB. Lastly, the small sample size of reclassified patients may be underpowered to detect a difference in prognostic discrimination between the AJCC 8th and 7th editions, and the overall low representation of patients with N2 disease (approximately 2.5%) in the NCDB database [32] may have affected the statistical association of N2 disease and death in patients with T4 stage.

## 4. Materials and Methods

### 4.1. Data Sources and Samples

Patients with GBC were identified from the NCDB between 2004 and 2015 using ICD-O-3 topography code C23.9 and histology codes 8140, 8141, 8144, 8201, 8210, 8211, 8255, 8260–63, 8310, and 8323. T classification was identified based on CS_Extension coding for both the 8th and 7th AJCC staging. N classification was identified through the CS_Lymph_Nodes coding for AJCC 7th, whereas AJCC 8th edition N classification was based on the number of positive lymph nodes (RX Sum—Scope_Reg_LN_Sur_(2003+) and the regional node positivity code (Regional_Nodes_Positive). M staging for both AJCC classifications was extracted using CS metastasis codes (CS_Mets_At_Dx and CS_Mets_Eval).

### 4.2. Statistical Analysis and Outcomes of Interest

GBC patients were classified according to both AJCC 8th and 7th classification systems. The discriminatory ability between AJCC 8th and 7th editions was evaluated with Harrell’s concordance index (c-index) [53,54]. Further, the total cohort was stratified by T stage to examine the effect of nodal status on survival. In addition, a multiple Cox proportional hazards regression analysis was used to identify factors associated with survival according to the AJCC 8th edition T-stage. The model was adjusted for age group, sex, race, year of diagnosis, insurance status, Charlson–Deyo score, tumor grade, tumor stage, extent of surgery, facility type, center volume, and surgical margin status. Scaled Schoenfeld residuals were visually examined for systematic variation over time to assess the proportional hazards assumption for categories of T or N staging, and specifically for all covariates included in adjusted models.

The aforementioned parameters were also evaluated with a univariate analysis (chi-squared test) to identify any association with reclassification (patients ≥IIIB stage), as well as a multiple Cox regression analysis to identify significant predictors for survival in patients with an available grade and stage ≥IIIB according to the AJCC 8th staging system.

All statistical analyses were performed with Stata 15 IC (StataCorp LLC, College Station, TX, USA). Two-sided *p*-values < 0.05 were used to determine statistical significance.

### 4.3. Data Availability Statement

The data used in this study are publicly available via the National Cancer Database (NCDB), available to investigators working within affiliated Commission on Cancer-accredited programs (https://m.facs.org/puf/). All patient data were de-identified and compliant with the Health Insurance Portability and Accountability Act of 1996 (HIPAA); patient consent was thus waived and the study was approved by the Duke University Medical Center Institutional Review Board.

## 5. Conclusions

In conclusion, the updated AJCC 8th GBC staging system was comparable to the 7th and no major improvements were identified in terms of GBC survival discrimination. In our analysis, patients who were classified in a higher stage with the AJCC 8th edition, age ≥70 years, higher Charlson–Deyo score, non-private insurance, and higher tumor grade were associated with worse prognosis. The recently implemented changes in N classification assessment do not appear to improve the prognostic performance of the AJCC cancer staging system, however, the risk of death was confirmed to be higher in patients with T1–T3 disease with N2 compared with N1 disease. Further prospective studies are needed to validate the T2 stage subclassification as well as to clarify the association, if any is actually present, between advanced N staging and increased risk of death in patients of the same T stage.

## Figures and Tables

**Table 1:** American Joint Committee on Cancer (AJCC) 8th vs. 7th classification staging criteria of patients with gallbladder cancer (GBC).

AJCC 8th	AJCC 7th	TNM 8th	Changes between AJCC 8th and 7th Editions
I	I	T1N0M0	Same criteria
IIA	II	T2aN0M0	IIA: Peritoneal side perimuscular connective tissue invasion
IIB		T2bN0M0	IIB: Hepatic side perimuscular connective tissue invasion
IIIA	IIIA	T3N0M0	N staging changed from a location-based to a number-based approach: N1: 1–3 regional nodes N2: ≥4 regional nodes
IIB	IIIB	T1–3N1M0	
IVA	IVA	T4N0-1M0	
IVB	IVB	TxN2M0 TxNxM1	

**Table 2:** (**a**) AJCC 8th vs. 7th classification of GBC patients from the National Cancer Database (NCDB). (**b**) GBC patients from the NCDB database: population characteristics.

(**a**)								
**AJCC 7th**		**AJCC 8th**						
		**I**	**II**	**IIIA**	**IIIB**	**IVA**	**IVB**	**Total**
I		1058	0	0	0	0	0	1058
II		0	2393	0	0	0	0	2393
IIIA		0	0	1000	0	0	0	1000
IIIB		0	0	0	1512	0	144	1656
IVA		0	0	0	0	163	14	177
IVB		0	0	0	43	1	1415	1459
Total		1058	2393	1000	1555	164	1573	7743
(**b**)								
				**Same Stage**		**Reclassified**		**Total**
Age	<55 years			861		33		894
	55–69 years			2631		76		2707
	70 and over			4049		93		4142
Sex	Male			2236		64		2300
	Female			5305		138		5443
Race	White			5844		163		6007
	Black			1097		27		1124
	Other			600		12		612
Charlson–Deyo	0			4980		132		5112
	1			1886		54		1940
	2+			675		16		691
Insurance	Private insurance			2130		68		2198
	Medicare/public, or uninsured			5411		134		5545
CenterVolume	5 or fewer cases/ 3 y period			4881		93		4974
	>5 cases/ 3 y period			2660		109		2769
Facility	Community			671		10		681
	Comprehensive community			2955		59		3014
	Academic			3915		133		4048
Year of diagnosis	2004–2006			1093		16		1109
	2007–2009			1283		32		1315
	2010–2012			2398		73		2471
	2013–2015			2767		81		2848
Surgery	Simple resection							
	Radical resection							
Chemotherapy	No			4830		82		4912
	Yes			2711		120		2831
Radiation	No			6162		121		6283
	Yes			1379		81		1460
Grade	Well-differentiated			1109		16		1125
	Moderate/ intermediate differentiation			3197		71		3268
	Poorly differentiated or undifferentiated			2745		99		2844
	N/A, unknown, high-grade dysplasia			490		16		506

**Table 3:** Impact of nodal disease on overall survival (OS) stratified by T-stage. CI, confidence interval.

N Stage	T1		T2		T3		T4	
	HR	95% CI	HR	95% CI	HR	95% CI	HR	95% CI
N1	1	[1.000,1.000]	1	[1.000,1.000]	1	[1.000,1.000]	1	[1.000,1.000]
N2	2.258	[1.656,3.079]	1.607	[1.455,1.778]	1.306	[1.178,1.449]	0.857	[0.603,1.218]
N+	1.928	[0.618,6.016]	1.643	[1.239,2.180]	1.393	[1.124,1.726]	1.062	[0.638,1.769]
Nx	1.281	[1.008,1.627]	2.221	[1.983,2.487]	2.194	[1.946,2.473]	0.991	[0.650,1.511]

**Table 4:** Factors associated with risk of death in GBC patients stratified by AJCC 8th T-stage classification.

Factors	Variable	T1		T2		T3		T4	
		HR	95% CI	HR	95% CI	HR	95% CI	HR	95% CI
Age	<55 years	1	[1.000,1.000]	1	[1.000,1.000]	1	[1.000,1.000]	1	[1.000,1.000]
	55–69 years	1.327	[0.861,2.044]	1.163	[0.967,1.398]	1.034	[0.888,1.203]	0.955	[0.598,1.525]
	70 and over	2.647	[1.740,4.025]	1.486	[1.235,1.787]	1.141	[0.974,1.337]	1.269	[0.766,2.101]
Sex	Male	1	[1.000,1.000]	1	[1.000,1.000]	1	[1.000,1.000]	1	[1.000,1.000]
	Female	0.748	[0.609,0.919]	0.934	[0.846,1.031]	0.960	[0.872,1.058]	0.817	[0.577,1.157]
Race	White	1	[1.000,1.000]	1	[1.000,1.000]	1	[1.000,1.000]	1	[1.000,1.000]
	Black	0.98	[0.733,1.311]	1.161	[1.018,1.324]	1.104	[0.965,1.263]	0.997	[0.628,1.584]
	Other	1.079	[0.704,1.652]	0.796	[0.663,0.956]	0.877	[0.639,0.927]	1.153	[0.658,2.019]
Charlson–Deyo	0	1	[1.000,1.000]	1	[1.000,1.000]	1	[1.000,1.000]	1	[1.000,1.000]
	1	0.995	[0.788,1.256]	1.149	[1.035,1.275]	1.136	[1.024,1.261]	0.965	[0.661,1.409]
	2+	1.510	[1.129,2.020]	1.573	[1.366,1.812]	1.126	[0.950,1.335]	0.691	[0.340,1.404]
Insurance	Private insurance	1	[1.000,1.000]	1	[1.000,1.000]	1	[1.000,1.000]	1	[1.000,1.000]
	Medicare/public, or uninsured	1.288	[0.966,1.717]	1.154	[1.027,1.296]	1.138	[1.018,1.272]	1.260	[0.895,1.772]
Center Volume	5 or fewer cases/ 3 y period	1	[1.000,1.000]	1	[1.000,1.000]	1	[1.000,1.000]	1	[1.000,1.000]
	>5 cases/ 3 y period	0.937	[0.738,1.188]	0.890	[0.801,0.988]	0.963	[0.871,1.065]	0.745	[0.538,1.031]
Facility	Community	1	[1.000,1.000]	1	[1.000,1.000]	1	[1.000,1.000]	1	[1.000,1.000]
	Comprehensive community	0.848	[0.614,1.171]	0.905	[0.772,1.060]	0.997	[0.843,1.179]	1.347	[0.674,2.695]
	Academic	0.780	[0.557,1.090]	0.792	[0.672,0.933]	0.760	[0.641,0.902]	1.132	[0.553,2.315]
Year of diagnosis	2004–2006	1	[1.000,1.000]	1	[1.000,1.000]	1	[1.000,1.000]	1	[1.000,1.000]
	2007–2009	0.954	[0.707,1.286]	1.061	[0.905,1.243]	1.007	[0.922,1.152]	1.639	[1.039,2.586]
	2010–2012	0.750	[0.572,0.982]	0.783	[0.678,0.904]	0.760	[0.661,0.873]	1.493	[0.939,2.374]
	2013–2015	0.489	[0.364,0.657]	0.745	[0.645,0.861]	0.728	[0.634,0.836]	1.044	[0.636,1.713]
Surgery	Simple resection	1	[1.000,1.000]	1	[1.000,1.000]	1	[1.000,1.000]	1	[1.000,1.000]
	Radical resection	0.923	[0.728,1.172]	0.957	[0.855,1.070]	0.915	[0.816,1.026]	0.836	[0.554,1.261]
Chemotherapy	No	1	[1.000,1.000]	1	[1.000,1.000]	1	[1.000,1.000]	1	[1.000,1.000]
	Yes	1.043	[0.693,1.569]	1.003	[0.894,1.127]	0.69	[0.622,0.764]	0.637	[0.446,0.910]
Radiation	No	1	[1.000,1.000]	1	[1.000,1.000]	1	[1.000,1.000]	1	[1.000,1.000]
	Yes	0.832	[0.503,1.377]	0.638	[0.553,0.735]	0.619	[0.546,0.702]	0.515	[0.337,0.787]
Grade	Well- differentiated	1	[1.000,1.000]	1	[1.000,1.000]	1	[1.000,1.000]	1	[1.000,1.000]
	Moderate/ intermediate differentiation	1.218	[0.951,1.560]	1.535	[1.313,1.795]	1.176	[0.970,1.426]	1.610	[0.807,3.212]
	Poorly differentiated or undifferentiated	1.968	[1.460,2.654]	2.434	[2.080,2.849]	1.765	[1.460,2.133]	1.822	[0.926,3.583]
	N/A, unknown, high-grade dysplasia	1.264	[0.909,1.757]	1.730	[1.348,2.221]	1.550	[1.202,2.000]	1.504	[0.670,3.380]
N stage	N0	1	[1.000,1.000]	1	[1.000,1.000]	1	[1.000,1.000]	1	[1.000,1.000]
	N1	2.416	[1.683,3.467]	1.74	[1.554,1.947]	1.450	[1.304,1.612]	1.095	[0.729,1.645]
	N2	2.243	[0.665,7.566]	1.775	[1.331,2.369]	1.527	[1.228,1.898]	1.693	[0.949,3.021]
	Nx	1.256	[0.978,1.612]	2.097	[1.866,2.356]	2.010	[1.777,2.274]	1.100	[0.680,1.780]

**Table 5:** Factors associated with risk of death in GBC patients with stage ≥ IIIB.

Factors	Variable	AJCC 7th Edition		AJCC 8th Edition	
		HR	95% CI	HR	95% CI
Stage	Stage IIIB	1	[1.000,1.000]	1	[1.000,1.000]
	Stage IVA	1.968	[1.638,2.365]	1.688	[1.481,1.923]
	Stage IVB	2.379	[2.120,2.670]	2.008	[1.878,2.148]
Age	<55 years	1	[1.000,1.000]	1	[1.000,1.000]
	55–69 years	0.994	[0.867,1.139]	0.982	[0.857,1.126]
	70 and over	1.139	[0.986,1.316]	1.111	[0.963,1.283]
Sex	Male	1	[1.000,1.000]	1	[1.000,1.000]
	Female	0.899	[0.821,0.985]	0.917	[0.838,1.003]
Race	White	1	[1.000,1.000]	1	[1.000,1.000]
	Black	0.997	[0.885,1.123]	1.005	[0.893,1.131]
	Other	0.862	[0.734,1.013]	0.856	[0.729,1.005]
Charlson–Deyo	0	1	[1.000,1.000]	1	[1.000,1.000]
	1	1.07	[0.974,1.176]	1.065	[0.970,1.170]
	2+	1.347	[1.159,1.566]	1.313	[1.131,1.524]
Insurance	Private insurance	1	[1.000,1.000]	1	[1.000,1.000]
	Medicare/public, or uninsured	1.098	[0.996,1.211]	1.093	[0.992,1.205]
Facility type	Community	1	[1.000,1.000]	1	[1.000,1.000]
	Comprehensive community	0.974	[0.837,1.134]	0.996	[0.856,1.158]
	Academic	0.759	[0.650,0.886]	0.768	[0.658,0.897]
Center Volume	5 or fewer cases/3 y period	1	[1.000,1.000]	1	[1.000,1.000]
	>5 cases/3 y period	0.932	[0.851,1.021]	0.919	[0.840,1.007]
Year of diagnosis	2004–2006	1	[1.000,1.000]	1	[1.000,1.000]
	2007–2009	1.008	[0.883,1.150]	1.009	[0.884,1.152]
	2010–2012	0.956	[0.842,1.085]	0.949	[0.836,1.076]
	2013–2015	0.882	[0.776,1.003]	0.869	[0.766,0.986]
Surgery	Simple resection	1	[1.000,1.000]	1	[1.000,1.000]
	Radical resection	0.938	[0.846,1.040]	0.931	[0.840,1.032]
Chemotherapy	No	1	[1.000,1.000]	1	[1.000,1.000]
	Yes	0.579	[0.529,0.634]	0.57	[0.521,0.624]
Radiotherapy	No	1	[1.000,1.000]	1	[1.000,1.000]
	Yes	0.756	[0.676,0.846]	0.74	[0.661,0.827]
Grade	Well-differentiated	1	[1.000,1.000]	1	[1.000,1.000]
	Moderate/intermediate differentiation	1.43	[1.194,1.712]	1.446	[1.210,1.727]
	Poorly differentiated or undifferentiated	1.953	[1.636,2.332]	1.958	[1.644,2.334]
	N/A, unknown, high-grade dysplasia	1.54	[1.216,1.949]	1.498	[1.185,1.894]
Nodal disease	N0	1	[1.000,1.000]	1	[1.000,1.000]
	N1	1.224	[1.067,1.404]	1.139	[0.995,1.304]
	N2	0.853	[0.682,1.066]	0.861	[0.713,1.039]
	NX	1.578	[1.371,1.816]	1.535	[1.336,1.764]

## Data Availability

The data that used in this study are publicly available via the National Cancer Database (NCDB), which are available to investigators working within affiliated Commission on Cancer-accredited programs.
